# Supplementary material for: Crosslinking-induced patterning of MOFs by direct photo- and electron-beam lithography
Source: Nat Commun. 2024 Apr 4;15:2920. doi: 10.1038/s41467-024-47293-6 (PMC10995132; doi:10.1038/s41467-024-47293-6)
Supplement: Supplementary file 1 — Supplementary Information [file 41467_2024_47293_MOESM1_ESM.pdf]

SUPPLEMENTARY INFORMATION

Crosslinking-induced patterning of MOFs by direct photo- and electron-beam lithography

Xiaoli Tian,[1] Fu Li,[1,2] Zhenyuan Tang,[2] Song Wang,[1] Kangkang Weng,[1] Dan Liu,[1] Shaoyong Lu,[1] Wangyu Liu,[1] Zhong Fu,[1] Wenjun Li,[1] Hengwei Qiu,[1] Min Tu,[2] Hao Zhang,[1] Jinghong Li[1,3,4]*

[1] Department of Chemistry, Center for Bioanalytical Chemistry, Key Laboratory of Bioorganic Phosphorus Chemistry & Chemical Biology (Ministry of Education), Tsinghua University, Beijing 100084, China.

[2] Shanghai Institute of Microsystem and Information Technology, Chinese Academy of Sciences, Shanghai 200050, China.

[3] Beijing Institute of Life Science and Technology, Beijing 102206, China.

[4] Center for Bioanalytical Chemistry, Hefei National Laboratory of Physical Science at Microscale, University of Science and Technology of China, Hefei 230026, China.

[*] Email: hzhangchem@mail.tsinghua.edu.cn

Supplementary Information includes

Supplementary Tables 1 to 3

Supplementary Figures 1 to 26

Supplementary References

Supplementary Tables and Figures

Supplementary Table 1. Summary of representative MOF patterning methods.

Supplementary Table 2. Details on the colloidal MOFs and parameters for their direct photolithography via CLIP-MOF.

Supplementary Table 3. Summary of BET surface area, pore volume, and pore size of various MOF NP powders before and after CLIP-MOF treatment.

Supplementary Fig. 1. TEM images of various colloidal MOF NPs.

Supplementary Fig. 2. Synthetic procedures and NMR spectra of bisPFPA crosslinkers.

Supplementary Fig. 3. Photographs of ZIF-8@BrijC10 NPs in methanol with bisPFPA crosslinkers before and after 254 nm UV exposure.

Supplementary Fig. 4. Characterizations of ZIF-8@BrijC10.

Supplementary Fig. 5. FTIR spectra of thin films composed of ZIF-8@BrijC10 and bisPFPA with different UV doses.

Supplementary Fig. 6. Optical images of microarrays of ZIF-8@BrijC10 patterns via direct photolithography.

Supplementary Fig. 7. Optical images of ZIF-8@BrijC10 patterns with different UV doses.

Supplementary Fig. 8. SEM images of ZIF-8@BrijC10 patterns with different mass fraction of bisPFPA.

Supplementary Fig. 9. Height profiles, LER analysis, and AFM images of patterned ZIF-8@BrijC10 films via direct photolithography.

Supplementary Fig. 10. Additional optical image of the large-area pattern of ZIF-8@BrijC10 in the format of “the periodic table of elements”.

Supplementary Fig. 11. Optical images of patterned ZIF-8@BrijC10 films on different substrates.

Supplementary Fig. 12. Optical images of patterns of ZIF-8@BrijC10 after 24 h immersion in different solvents.

Supplementary Fig. 13. Film retention or contrast curve of ZIF-8@BrijC10 films at different UV doses.

Supplementary Fig. 14. Film retention of ZIF-8@BrijC10 MOF films versus aging days.

Supplementary Fig. 15. SEM images of various MOF films patterned by CLIP-MOF.

Supplementary Fig. 16. Optical images and EDS mapping of binary and ternary MOF patterns.

Supplementary Fig. 17. Optical images and height profiles of binary cross-line patterns of different MOFs.

Supplementary Fig. 18. Preserved crystallinity of pristine and patterned HKUST-1@OA /OLAM and Eu(BTC)@PVP films.

Supplementary Fig. 19. XRD patterns of films of pristine and patterned MOFs collected after UV exposure with different doses.

Supplementary Fig. 20. HRTEM images and EDS data of pristine and CLIP-MOF treated ZIF-8@CTAB films.

Supplementary Fig. 21. BJH size distribution of pristine and CLIP-MOF treated MOF NP powders.

Supplementary Fig. 22. FTIR Spectra of MOF films during CLIP-MOF processes.

Supplementary Fig. 23. Scheme and responses of the patterned ZIF-8@BrijC10 diffraction grating sensors towards acetone vapors.

Supplementary Fig. 24. Responses and SEM images of the patterned ZIF-8@BrijC10 diffraction grating sensors during multi-cycle utilization.

Supplementary Fig. 25. Cyclic voltammetry curve of the electrochromic ZIF-8@BrijC10 film with NDI molecules.

Supplementary Fig. 26. Additional images of MOF patterns made via direct e-beam lithography.

Supplementary Table 1. Summary of representative MOF patterning methods.

Method	MOF	Resolution	Features
This work	various colloidal MOFs (ZIF-8, ZIF-7, HKUST-1, UiO-66, Eu(BTC))	5 µm (photolithography, defined by the mask resolution) 70 nm (e-beam lithography)	-direct, resist-free; -multimaterial; -multilayered; -scalable, large area (photolithography); -low UV or e-beam doses; -nondestructive.
Direct e-beam/ X-ray lithography	ZIFs with halogenated linkers (ZIF-71, ZIF-72, and ZIF-8)[1]	≈10 µm (X-ray lithography) sub-50-nm (e-beam lithography)	-direct, resist-free; -limited scope of patternable MOFs (with halogenated linkers); -single-layered; -hard to be scalable; -high e-beam doses; -nondestructive.
	Cu_2L_2DABCO with Br-containing linkers[2]	≈10 µm (X-ray lithography)	
	ZIF-L[3], ZIF-8 and MIL-101[4]	≈30 nm (e-beam lithography on a single ZIF-L nanocrystal)	
Patterning of metal oxides and subsequent conversion to MOF patterns	Cu-BHT[5]	≈1 µm (photolithography of CuO films with photo resist)	-resist-based or resist-free patterning of MOF precursors (metal oxides); -limited scope of patternable MOFs; -incomplete metal oxide-MOF conversion; -hard to achieve multimaterial, multilayered patterning.
	ZIF-8 and ZIF-67[6]	≈200 nm (direct e-beam writing on ZmIm-sensitized ZnO films)	
	ZIF-8[7]	hand-writing (5 mm), laser (70 µm), focused beam (270 nm) patterning of ZnO films	
	HKUST-1[8]	≈125 µm (patterning of Cu electrodes, followed by conversion to $Cu(OH)_2$ and then Cu MOFs)	

Photo- and X-ray lithography with resist	ZIF-8[9]	≈10 µm (photolithography with photoresist)	-require resist;<br>-scalable, large area;<br>-potential blockage or damage of the pore properties of MOFs by resists or etching procedures;<br>-composites of MOF/resists (instead of only MOFs) are patterned in some cases.
	ZIF-9/sol-gel composite[10]	≈5 µm (X-ray lithography with phenyltriethoxysilane)	
	HKUST-11[1]	≈500 µm (photolithography with a small volume of HKUST-1 precursor solution and photobase generator)	
Nanoimprinting and growth/ assembly of MOFs with pre-patterned polymer templates	NH_2-MIL-53, ZIF-67, and ZIF-8[12]	≈10 µm (imprinting MOF films on photolithography patterned SU-8 stamps)	-require pre-patterned stamps/templates;<br>-poorly defined pattern edges;<br>-not scalable;<br>-low resolution.
	colloidal ZIF-8[13]	≈100 nm (nanoimprinting/ assembly colloidal ZIF-8 with pre-patterned PDMS stamps)	
	HKUST-1, MOF-5, and ZIF-8[14]	≈10 µm (nucleation and growth of MOF crystals directed by pre-patterned PDMS stamps)	
	UiO-66[15]	≈50 µm (in-situ growth of UiO-66 membranes on pre-patterned YSZ ceramic stamps)	
Inkjet/Aerosol Jet Printing	HKUST-1[16]	≈200 µm	-low cost;<br>-scalable, large area;<br>-nondestructive;<br>-low resolution.
	UTSA-280[17]	≈100 µm (estimated)	
Evaporation-directed crack-patterning	colloidal MIL-101 and colloidal ZIF-8[18]	16 µm	-low cost;<br>-scalable, large area;<br>-limited types of patterns (periodic lines with random cracks)
Directed growth/ assembly of MOFs on chemically	MOF-5[19]	≈40 µm (size of films)	-chemically defined substrates with self-assembled organic monolayers by AFM related techniques;
	HKUST-1[20]	≈1 µm (size of single crystals)	
	HKUST-1[21]	≈3 µm	

functionalized substrates		(size of SURMOF films)	-growth of MOF films or crystals from solution of precursors;<br>-limited control of patterned MOF film quality or crystal size uniformity.
	UiO-66 and MIL-101[22]	≈50 μm	-chemically defined substrates with alkyne- and carboxylic acid-terminated SAMs;<br>-capable to pattern two different MOFs.
Directed growth/ assembly of MOFs on electrically defined substrates	HKUST-1[23]	≈100 μm	-growth or assembly of MOFs on prepatterned electrodes;<br>-low resolution and limited pattern fidelity;<br>-limited scope of patternable MOFs.
	UiO-66 and NU-100[24]	≈25 μm	

Supplementary Table 2. Details on the colloidal MOFs and parameters for their direct photolithography via CLIP-MOF.

MOFs	Ligand[†]	Solvent	Exposure dose (mJ cm^{-2})	Developer[*]	Mass ratio[‡] (wt%)	Developing time
ZIF-8@BrijC10	BrijC10	methanol	90	water + methanol	10<br>5<br>1	5 min<br>20 min<br>20 min
ZIF-8@CTAB	CTAB	methanol	75	water + methanol	5	30 min
ZIF-7@PEI	PEI	methanol	120	water + methanol	8	5 min
HKUST-1@OA/OLAM	OA/OLAM	toluene	60	toluene	5	30 s
HKUST-1@OA	OA	toluene	120	toluene	10	1 min
UiO-66@PAA	PAA	methanol	150	water + methanol	20	1 min
Eu(BTC)@PVP	PVP	methanol	120	water + methanol	20	5 min

[†] BrijC10, polyethylene glycol hexadecyl ether; CTAB, hexadecyl trimethyl ammonium bromide; PEI, polyetherimide; OA, oleic acid; OLAM, oleylamine; PAA, polyacrylic acid; PVP, polyvinyl pyrrolidone; BTC, benzene-1,3,5-tricarboxylic acid.

[*] water + methanol: 1:1 in volume.

[‡] Mass ratio, bisPFPA: MOF NPs.

Supplementary Table 3. Summary of BET surface area, pore volume, and pore size of various MOF NP powders before and after CLIP-MOF treatment.*

**MOFs**	**BET surface area (cm^2 g^{-1})**	**total pore volume (cm^2 g^{-1})**	**micropore volume (cm^2 g^{-1})**	**pore diameter (nm)**
ZIF-8@BrijC10	999.5	0.95	0.44	0.58
ZIF-8@BrijC10 CLIP-MOF	887.7	0.82	0.38	0.57
reference data[25]	1,478.5	–	0.58	–
ZIF-8@CTAB	1,721.3	1.22	0.63	0.87
ZIF-8@CTAB CLIP-MOF	1,660.3	1.16	0.61	0.87
reference data[26]	1,200	–	0.70	–
UiO-66@PAA	1,209	1.13	0.49	0.57
UiO-66@PAA CLIP-MOF	1,066	0.96	0.44	0.56
reference data[27]	1,050	–	–	–
HKUST-1@OA	1,400	1.11	0.57	0.43
HKUST-1@OA CLIP-MOF	1,479	1.32	0.60	0.47
reference data[28]	1,472	–	–	0.78

*Reference data for each MOF show porosity data for MOF NPs synthesized in the same or similar approach and with similar sizes and ligands in previous reports.

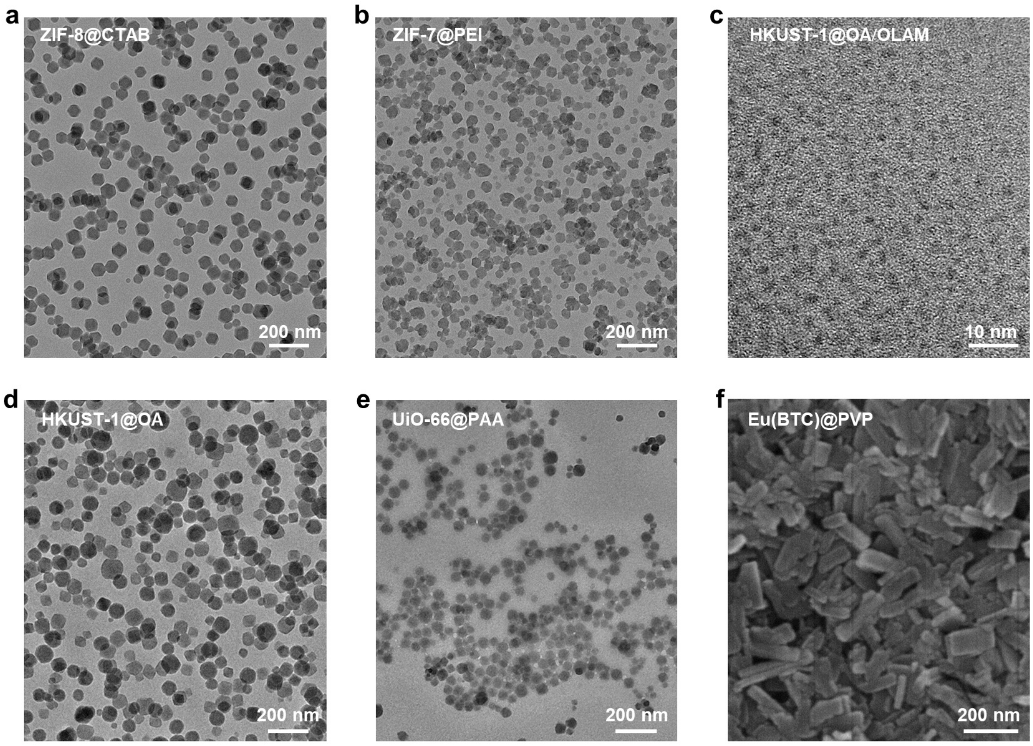

Supplementary Figure 1. TEM images of various colloidal MOF NPs. a ZIF-8@CTAB, **b** ZIF-7@PEI, **c** HKUST-1@OA/OLAM, **d** HKUST-1@OA, **e** UiO-66@PAA, **f** Eu(BTC)@PVP.

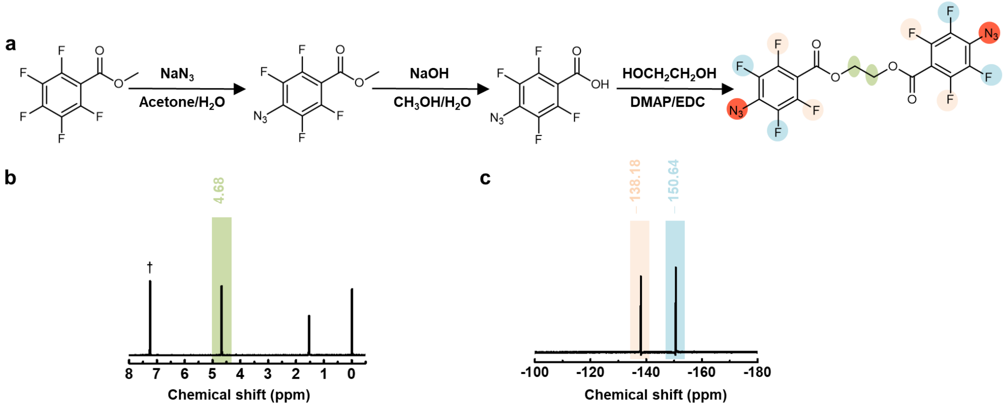

Supplementary Figure 2. Synthetic procedures and NMR spectra of bisPFPA crosslinkers. a Synthesis of bisPFPA crosslinkers. **b** ^{1}H and **c** ^{19}F-NMR spectra of bisPFPA in $CDCl_3$. Labeled (†) is the resonance of $CDCl_3$ solvent. The color shades and numbers marked in **b**, **c** indicate the resonance and chemical shifts of H or F atoms indicated by the same color in **a**.

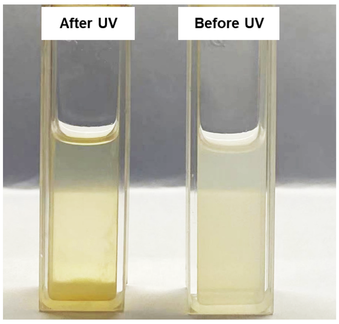

Supplementary Figure 3. Photographs of ZIF-8@BrijC10 NPs in methanol with bisPFPA crosslinkers before and after 254 nm UV exposure. ZIF-8@BrijC10 NPs lose their colloidal stability after UV exposure due to the crosslinking, forming precipitates at the bottom of the cuvette.

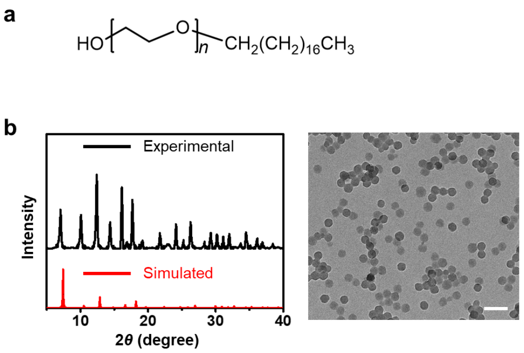

Supplementary Figure 4. Characterizations of ZIF-8@BrijC10. **a** Chemical structure of BrijC10 ligands (n ≈10, averaged molecular weight ≈683). **b** Simulated (red traces) and measured (black traces) XRD patterns and TEM image of ZIF-8@BrijC10 NPs. Scale bar, 100 nm.

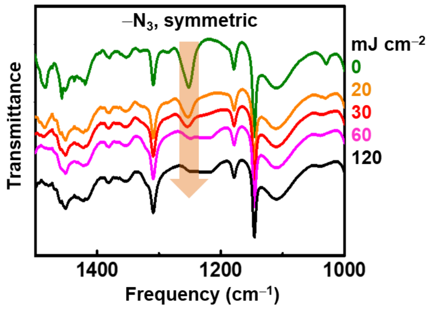

Supplementary Figure 5. FTIR spectra of thin films composed of ZIF-8@BrijC10 and bisPFPA with different UV doses. Data plots in different colors correspond to films exposed with different doses. Changes in the intensity of symmetric $–N_3$ (≈1250 cm^{-1}) vibrational modes are highlighted by the orange arrow.

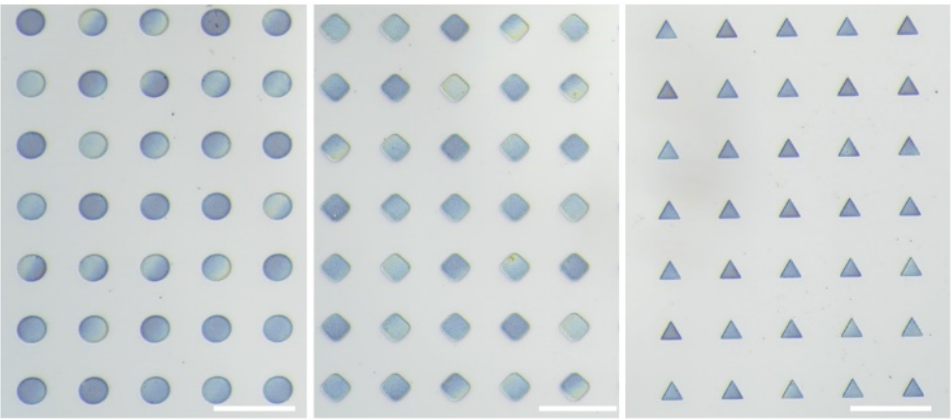

Supplementary Figure 6. Optical images of microarrays of ZIF-8@BrijC10 patterns via direct photolithography. Scale bars, 100 μm.

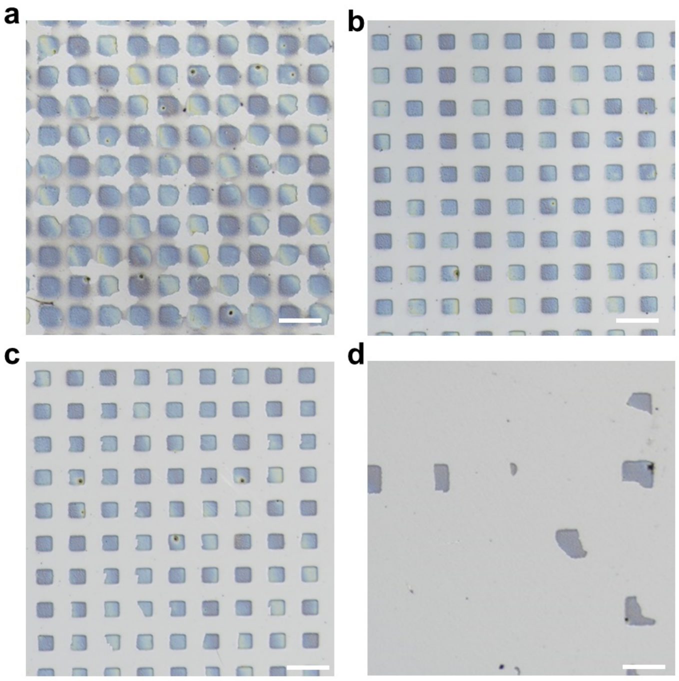

Supplementary Figure 7. Optical images of ZIF-8@BrijC10 patterns with different UV doses. **a**120, **b** 90, **c** 60, **d** 30 mJ cm^{-2}. Exposure of 60 or 90 mJ cm^{-2} led to high quality patterns. Patterning with significantly higher or lower UV doses produce expanded or fragmentary patterns. Scale bars, 100 μm.

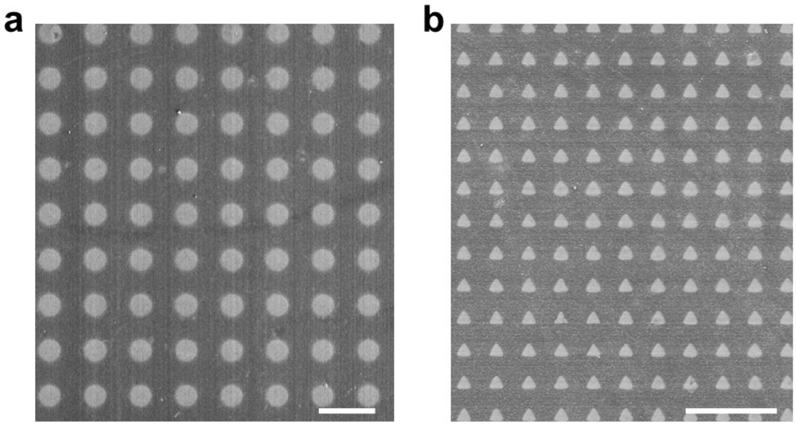

Supplementary Figure 8. SEM images of ZIF-8@BrijC10 patterns with different mass fraction of bisPFPA. a **≈**5 wt%, **b** **≈**1 wt%, to the mass of MOFs. Scale bars, 200 μm.

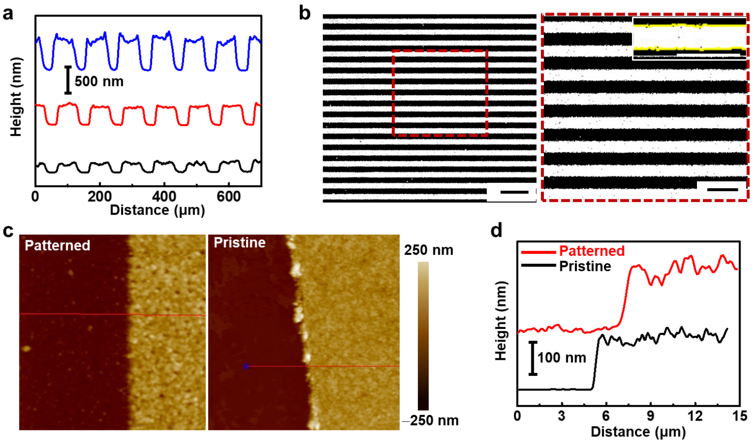

Supplementary Figure 9. Height profiles, LER analysis, and AFM images of patterned ZIF-8@BrijC10 films via direct photolithography. a Height profiles of line patterns of ZIF-8@BrijC10 with different thickness. **b** LER analysis of patterned films (LER ≈113 nm). The edge of line was extracted and analyzed by a software ImageJ. Scale bars, (left) 50 μm, (right) 25 μm, and (inset) 25 μm. **c, d** AFM images and height profiles of pristine (black curve) and patterned (red curve) films.

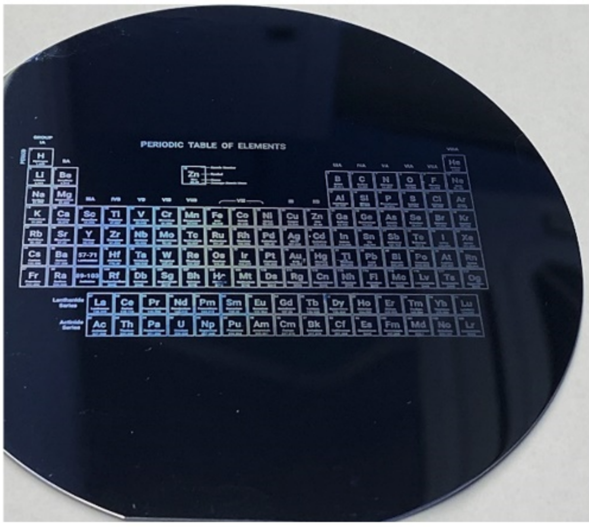

Supplementary Figure 10. Additional optical image of the large-area pattern of ZIF-8@BrijC10 in the format of “the periodic table of elements”. The size of the silicon wafer is 10 cm.

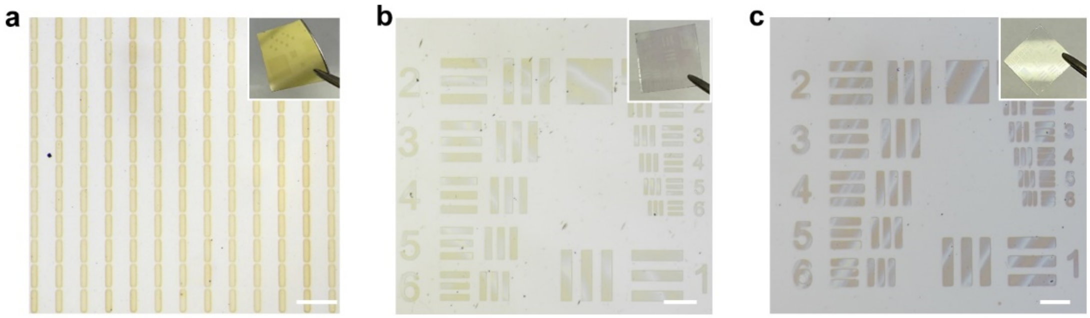

Supplementary Figure 11. Optical images of patterned ZIF-8@BrijC10 films on different substrates. **a** 30 nm Au film coated on silicon substrate, **b** ITO glass, **c** glass slide. Insets show corresponding photographs. Scale bars, 100 µm.

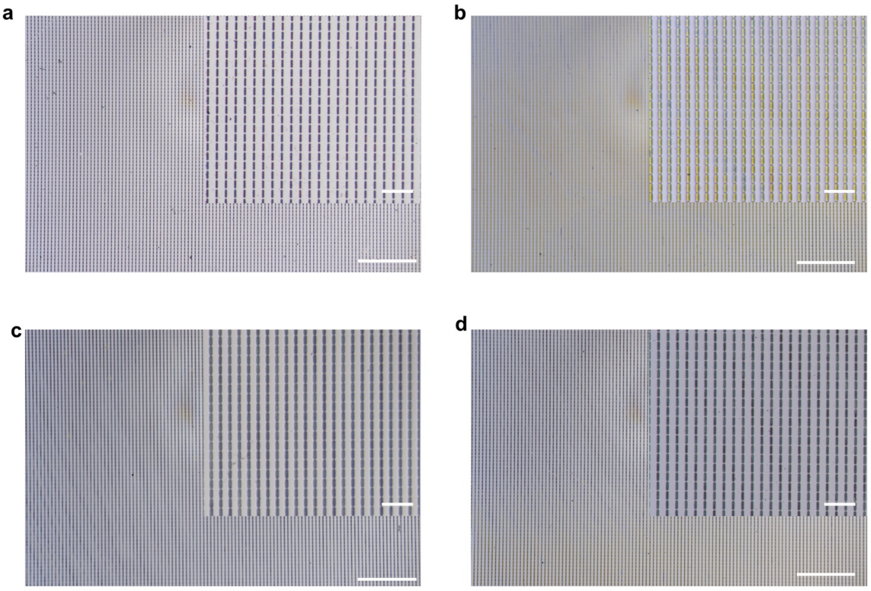

Supplementary Figure 12. Optical images of patterns of ZIF-8@BrijC10 after 24 h immersion in different solvents. a acetone, **b** ethanol, **c** toluene, **d** methanol. Scale bars, 500 μm, (insets) 100 μm. It is reported[29] the van der Waals attractive force between a single MOF NP and the flat substrate, at the separation of 1 nm, is about a few nN. The crosslinking between adjacent NPs in the patterns and the small separation between MOFs and substrates (<1 nm) further increase the adhesion. This contributes to the good stability of patterned MOF films against solvent soaking.

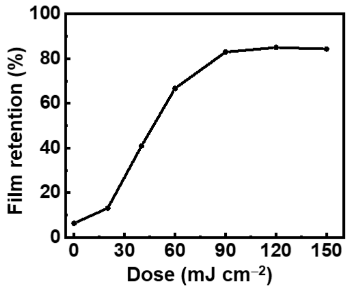

Supplementary Figure 13. Film retention or contrast curve of ZIF-8@BrijC10 films at different UV doses. A low dose of 60 mJ cm^{-2} results in over 60% film retention that subsequently saturates (≈83%) at 90 mJ cm^{-2}. The non-zero film retention for unexposed films may come from nonspecific adhesion of residual MOF NPs after solvent developing.

The contrast curve or film retention was measured via inductively coupled plasma-optical emission spectroscopy (ICP-OES) analysis of Zn. We exposed ZIF-8@BrijC10 NP films (exposure in the entire film) under different UV doses. After solvent developing, we collected the MOF NPs dissolved in the developer by evaporation. We then digested 1) the MOF NPs remaining in the film and 2) MOF NPs collected from the developer by using concentrated hydrochloric acid. By comparing the Zn concentration in these two digested solutions, we can calculate the ratio of MOF NPs remaining in the film after UV exposure and plot the film retention/contrast curve.

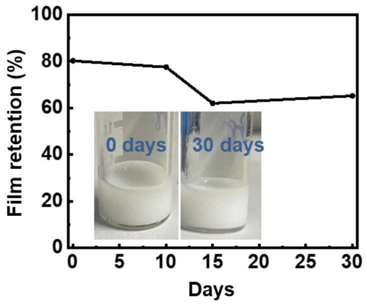

Supplementary Figure 14. Film retention of ZIF-8@BrijC10 MOF films versus aging days. Inset shows the photos of solutions containing ZIF-8@BrijC10 and bisazides after stored in dark for different days.

We monitored the film retention by using ICP-OES analysis with MOF NP solutions after stored for 0, 10, 15, and 30 days. When stored for 10 days, the film retention dropped very slightly from 80% to 78%, which is negligible considering the measurement errors. In comparison, patterning with a solution stored for 15 or 30 days showed a notably decreased film retention of 62%, even though there was no obvious destabilization of the solution (inset). The decreased patterning performance may come from the partial aggregation and the formation of small clusters of MOF NPs. These results suggest the aging time for ZIF-8@BrijC10 is about 10 days for best performance in patterning. In this work, colloidal MOFs and crosslinkers are stored separately and mixed at the time of use. Crosslinkers can be stored for more than six months and colloidal MOFs for more than one month, which is determined by the colloidal stability of MOF NP solutions and dependent on the NP sizes, ligands, concentration, and others.

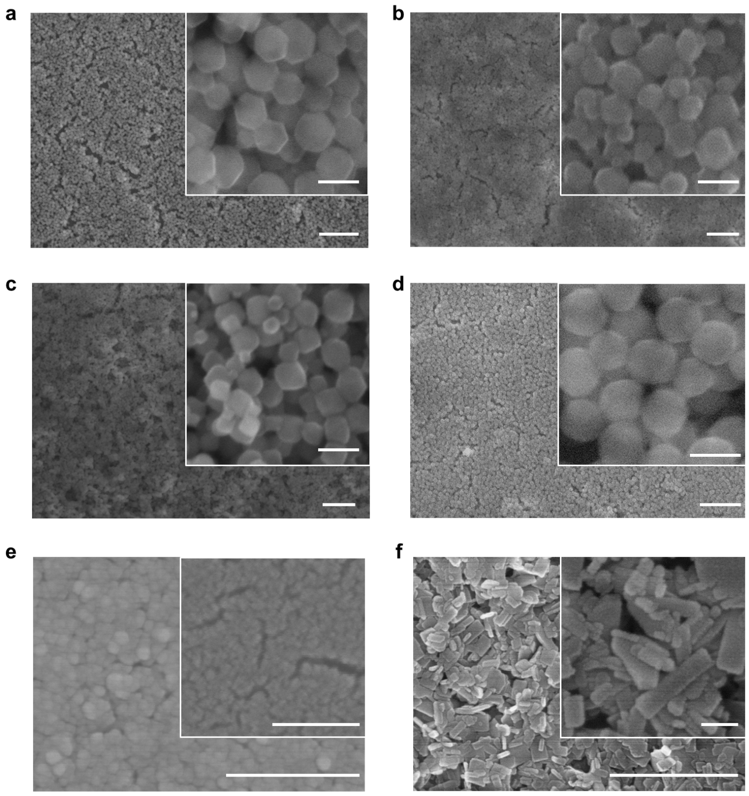

Supplementary Figure 15. SEM images of various MOF films patterned by CLIP-MOF. **a** ZIF-8@CTAB, **b** ZIF-7@PEI, **c** UiO-66@PAA, **d** HKUST-1@OA, **e** HKUST-1@OA/OLAM, **f** Eu(BTC)@PVP. The insets in each panel show the magnified view, highlighting the preservation of faceted structures of MOF NPs. Scale bars, 1 μm and (insets) 100 nm.

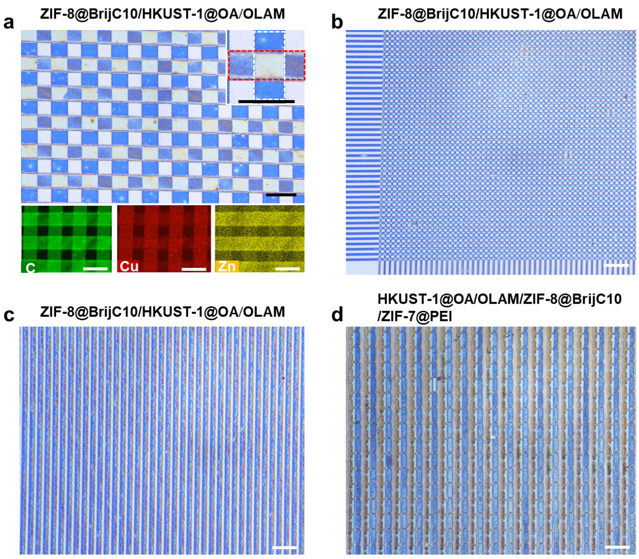

Supplementary Figure 16. Optical images and EDS mapping of binary and ternary MOF patterns. a Cross-line patterns of ZIF-8@BrijC10 and HKUST-1@OA/OLAM. The linewidth is 50 µm. Inset shows the magnified version highlighting the rectangles in dashed boxes (white, ZIF-8@BrijC10; red, HKUST-1@OA/OLAM). The bottom shows the corresponding EDS data. **b** Large-scale cross-line patterns of ZIF-8@BrijC10 and HKUST-1@OA/OLAM. The linewidth is 15 µm. **c** Large-scale patterns of alternating rectangles composed of ZIF-8@BrijC10 and HKUST-1@OA/OLAM. **d** Large-scale patterns containing three different MOFs (ZIF-8@BrijC10; HKUST-1@OA/OLAM; ZIF-7@PEI). Scale bars, 100 µm.

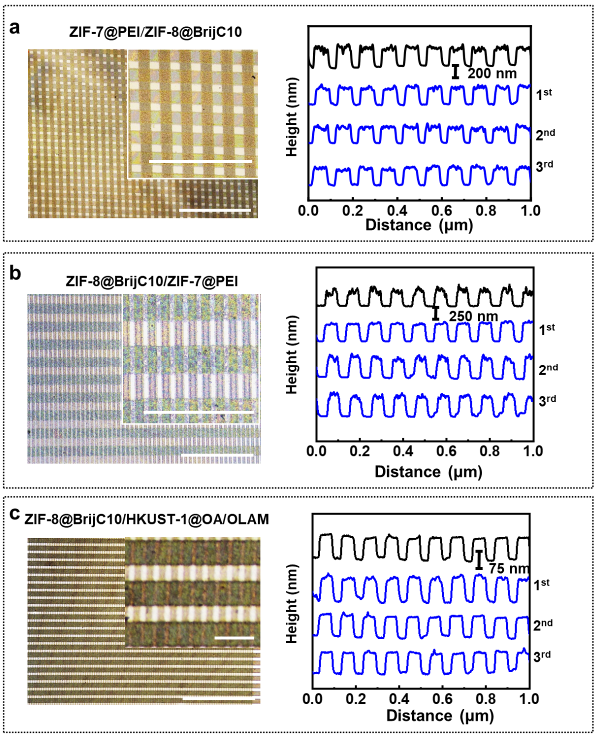

Supplementary Figure 17. Optical images and height profiles of binary cross-line patterns of different MOFs. a ZIF-7@PEI/ZIF-8@BrijC10, **b** ZIF-8@BrijC10/ZIF-7@PEI, **c** ZIF-8@BrijC10/HKUST-1@OA/OLAM. In each case, the MOF written on the left was firstly patterned as lines, followed by the patterning of the second layer (those written on the right). The heights of the first MOF layer were measured before (the black traces) and after (the blue traces) the patterning of the second layer. The heights of the first layer after the second layer patterning were measured at three different locations. Scale bars, 1 mm, and (insets) 500 µm.

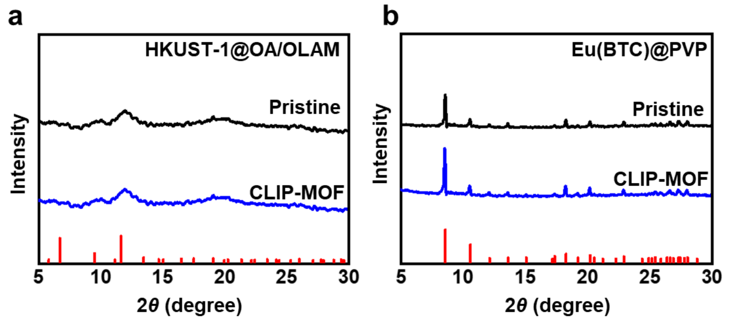

Supplementary Figure 18. Preserved crystallinity of pristine and patterned HKUST-1@OA/OLAM and Eu(BTC)@PVP films. **a** HKUST-1@OA/OLAM, **b** Eu(BTC)@PVP. Data for patterned films are plotted as blue traces. For comparison, XRD patterns of pristine MOF films (black traces) and the standard diffraction patterns (indicated by red vertical lines at the bottom) are included. Note that the relatively broad peaks of HKUST-1@OA/OLAM are due to their small particle sizes (≈3 nm, as estimated by Scherrer equation).

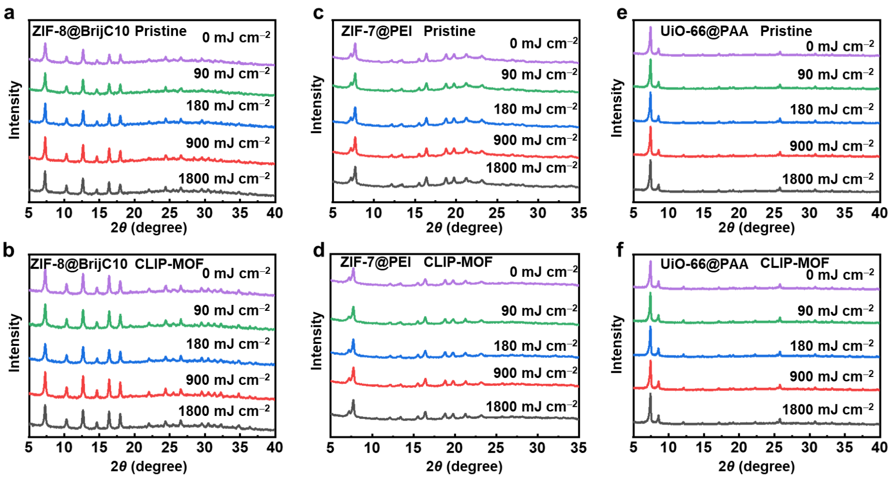

Supplementary Figure 19. XRD patterns of films of pristine and patterned MOFs collected after UV exposure with different doses. **a** ZIF-8@BrijC10, **b** ZIF-8@BrijC10 (CLIP-MOF), **c** ZIF-7@PEI, **d** ZIF-7@PEI (CLIP-MOF), **e** UiO-66@PAA, **f** UiO-66@PAA (CLIP-MOF). Data shown in different colors are collected on samples exposed with different UV doses. Note that regular patterning requires UV doses of ≈90 mJ cm^{-2}. UV exposure does not cause notable changes in the crystal structures of MOFs.

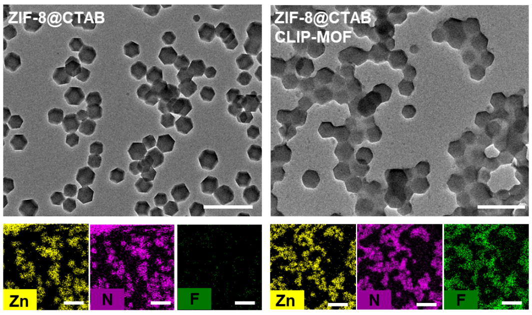

Supplementary Figure 20. HRTEM images and EDS data of pristine and CLIP-MOF treated ZIF-8@CTAB films. Scale bars, 500 nm.

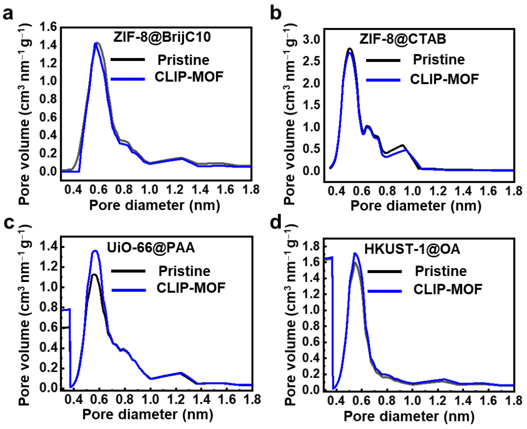

Supplementary Figure 21. BJH size distribution of pristine and CLIP-MOF treated MOF NP powders. a ZIF-8@BrijC10, **b** ZIF-8@CTAB, **c** UiO-66@PAA, **d** HKUST-1@OA. Black curves, pristine samples; blue curves, CLIP-MOF treated samples.

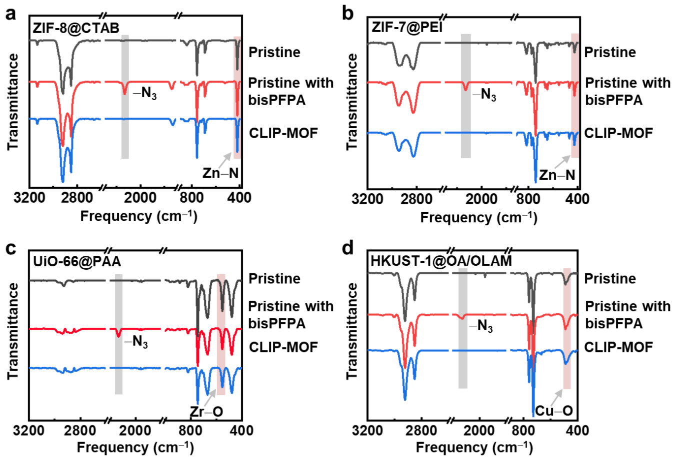

Supplementary Figure 22. FTIR Spectra of MOF films during CLIP-MOF processes. **a** ZIF-8@CTAB, **b** ZIF-7@PEI, **c** UiO-66@PAA, **d** HKUST-1@OA/OLAM. In each case, the spectra of pristine films (grey curves), films coated with bisazide-based crosslinkers (red curves), and films treated with CLIP-MOF procedures (addition of crosslinkers, UV exposure, and solvent developing, shown as blue curves) are compared. The color shades in each panel indicate the resonance peaks of $–N_3$ in the crosslinkers and metal–linker bonds in corresponding MOFs.

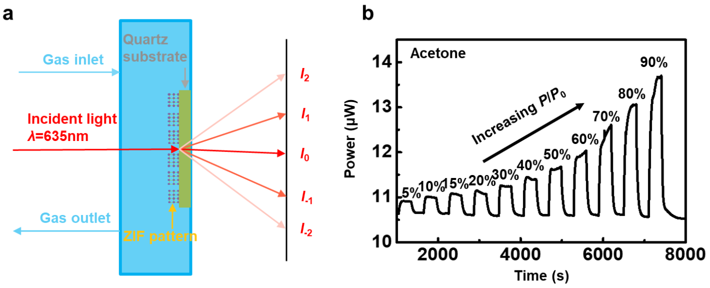

Supplementary Figure 23. Scheme and responses of the patterned ZIF-8@BrijC10 diffraction grating sensors towards acetone vapors. a Scheme of the set up. Changes in the light intensity of the first-order diffraction spot (I_1) were monitored. **b** Measurements of power during the changes in the relative vapor pressure of acetone (as indicated by the percentage numbers).

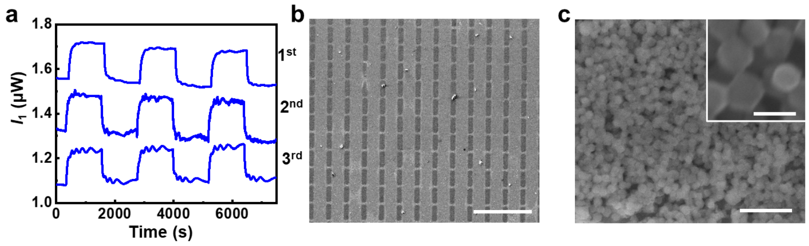

Supplementary Figure 24. Responses and SEM images of the patterned ZIF-8@BrijC10 diffraction grating sensors during multi-cycle utilization. a The optical power corresponding to the light intensity of the first-order diffraction spot in the 1st, 2nd, and 3rd cycle of measurement. The vapor pressure of ethanol for these measurements was 80%. **b, c** SEM images of the MOF patterns and the constituent MOF NPs in the diffraction grating sensor after 3 cycles of solvent immersion and gas vapor measurements. Scale bars, b 100 μm, c 500 nm, and inset in c 100 nm.

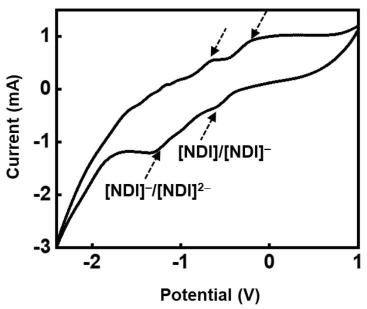

Supplementary Figure 25. Cyclic voltammetry curve of the electrochromic ZIF-8@BrijC10 film with NDI molecules. The arrows indicate the redox peaks of NDI.

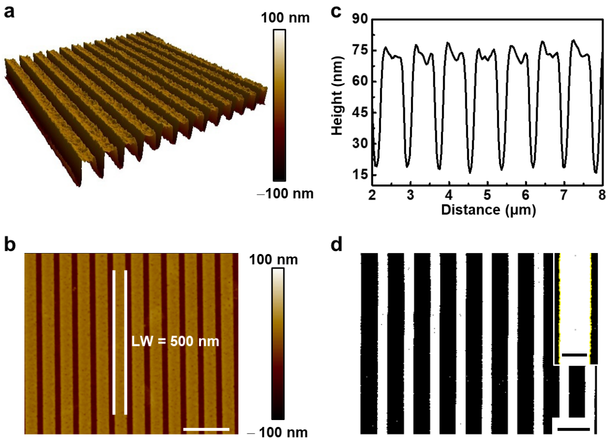

Supplementary Figure 26. Additional images of MOF patterns made via direct e-beam lithography. a–c 3D AFM, AFM and height profiles of patterns with 500 nm linewidth. **d** LER (≈1.5 nm) analysis of patterned HKUST-1@OA/OLAM films with 130 nm linewidth. The edge of line was extracted and analyzed by a software ImageJ. Scale bars, b 2 μm, d 500 nm, and inset in d, 100 nm.

Supplementary references

1. Tu, M. et al. Direct X-ray and electron-beam lithography of halogenated zeolitic imidazolate frameworks. *Nat. Mater.* **20**, 93–99 (2021).

2. de J. Velásquez-Hernández, M. et al. Fabrication of 3D oriented MOF micropatterns with anisotropic fluorescent properties. *Adv. Mater*. **35,** 2211478 (2023).

3. Miao, Y. & Tsapatsis, M. Electron beam patterning of metal-organic frameworks. *Chem. Mater.* **33**, 754–760 (2021).

4. Michael, T. et al. Controlling dissolution and transformation of zeolitic imidazolate frameworks by using electron-beam-induced amorphization. *Angew. Chem. Int. Ed.* **57**, 13592–13597 (2018).

5. Rubio-Giménez, V. et al. Chemical vapor deposition and high-resolution patterning of a highly conductive two-dimensional coordination polymer film. *J. Am. Chem. Soc.* **145**, 152–159 (2023).

6. Miao, Y. et al. Solvent-free bottom-up patterning of zeolitic imidazolate frameworks. *Nat. Commun.* **13**, 420 (2022).

7. Bo, R. et al. Paper-like writable nanoparticle network sheets for mask-less MOF patterning. *Adv. Funct. Mater.* **32**, 2100351 (2021).

8. Okada, K. et al. Copper conversion into $Cu(OH)_2$ nanotubes for positioning

$Cu_3(BTC)_2$ MOF crystals: Controlling the growth on flat plates, 3D architectures, and as patterns. *Adv. Funct. Mater.* **24**, 1969–1977 (2014).

9. Lu, G., Farha O.K., Zhang, W., Huo, F. & Hupp, J.T. Engineering ZIF-8 thin films for hybrid MOF-based devices. *Adv. Mater.* **24**, 3970–3974 (2012).

10. Dimitrakakis, C. et al. Top-down patterning of zeolitic imidazolate framework composite thin films by deep X-ray lithography. *Chem. Commun.* **48**, 7483–7485 (2012).

11. Keitz, B.K., Yu, C., Long, J. & Ameloot, R. Lithographic deposition of patterned metal-organic framework coatings using a photobase generator. *Angew. Chem. Int. Ed.* **53**, 5561–5565 (2014).

12. Doherty, C.M. et al. Combining UV lithography and an imprinting technique for patterning metal-organic frameworks. *Adv. Mater.* **25**, 4701–4705 (2013).

13. Dalstein, O. et al. Nanoimprinted, submicrometric, MOF-based 2D photonic structures: Toward easy selective vapors sensing by a smartphone camera. *Adv. Funct. Mater.* **26**, 81–90 (2016).

14. Ameloot, R. et al. Direct patterning of oriented metal-organic framework crystals via control over crystallization kinetics in clear precursor solutions. *Adv. Mater.* **22**, 2685–2688 (2010).

15. Huang, K., Wang, B., Guo, S. & Li, K. Micropatterned ultrathin MOF

membranes with enhanced molecular sieving property. *Angew. Chem. Int. Ed.* **57**, 13892–13896 (2018).

16. Zhuang, J., Ar, D., Yu, X., Liu, J. & Terfort. A. Patterned deposition of metal-organic frameworks onto plastic, paper, and textile substrates by inkjet printing of a precursor solution. *Adv. Mater.* **25**, 4631–4635 (2013).

17. Kravchenko, D.E. et al. Aerosol jet printing of the ultramicroporous calcium squarate metal-organic framework. *Chem. Mater.* **34**, 6809–6814 (2022).

18. Dalstein, O. et al. Evaporation-directed crack-patterning of metal-organic framework colloidal films and their application as photonic sensors. *Angew. Chem. Int. Ed.* **56**, 14011–14015 (2017).

19. Hermes, S., Schröder, F., Chelmowski, R., Wöll, C. & Fischer, R.A. Selective nucleation and growth of metal-organic open framework thin films on patterned COOH/CF_3-terminated self-assembled monolayers on Au (111). *J. Am. Chem. Soc.* **127**, 13744–13745 (2005).

20. Carbonell, C., Imaz, I. & Maspoch, D. Single-crystal metal-organic framework arrays. *J. Am. Chem. Soc.* **133**, 2144–2147 (2011).

21. Munuera, C., Shekhah, O., Wang, H., Wöll, C. & Ocal, C. The controlled growth of oriented metal-organic frameworks on functionalized surfaces as followed by scanning force microscopy. *Phys. Chem. Chem. Phys.* **10**, 7257–7261 (2008).

22. Semrau, A.L. et al. Selective positioning of nanosized metal-organic framework particles at patterned substrate surfaces. *Chem. Mater.* **32**, 9954–9963 (2020).

23. Ameloot, R. et al. Patterned growth of metal-organic framework coatings by electrochemical synthesis. *Chem. Mater.* **21**, 2580–2582 (2009).

24. Hod, I. et al. Directed growth of electroactive metal-organic framework thin films using electrophoretic deposition. *Adv. Mater.* **26**, 6295–6300 (2014).

25. Zhao, X., Fang, X., Wu, B., Zheng, L. & Zheng, N. Facile synthesis of size-tunable ZIF-8 nanocrystals using reverse micelles as nanoreactors. *Sci. China Chem.* **57**, 141–146 (2014).

26. Pan, Y. et al. Tuning the crystal morphology and size of zeolitic imidazolate framework-8 in aqueous solution by surfactants. *CrystEngComm* **13**, 6937–6940 (2011).

27. Morris, W. et al. Role of modulators in controlling the colloidal stability and polydispersity of the UiO-66 metal-organic framework. *ACS Appl. Mater. Inter* **9**, 33413–33418 (2017).

28. Cai, X., Xie, Z., Pang, M. & Lin, J. Controllable synthesis of highly uniform nanosized HKUST-1 crystals by liquid–solid–solution method. *Cryst. Growth Des.* **19**, 556–561 (2019).

29. J. N. Israelachvili, *Intermolecular and Surface Forces*. (ed. Third, 2011).
